# Synthesis and Physicochemical Characterization of Undecylenic Acid Grafted to Hyaluronan for Encapsulation of Antioxidants and Chemical Crosslinking

**DOI:** 10.3390/polym12010035

**Published:** 2019-12-24

**Authors:** Gloria Huerta-Ángeles, Martina Brandejsová, Kateřina Kopecká, František Ondreáš, Tomáš Medek, Ondrej Židek, Jaromír Kulhánek, Hana Vagnerová, Vladimir Velebný

**Affiliations:** Contipro a.s., Dolni Dobrouč 401, 561 02 Dolni Dobrouč, Czech Republic

**Keywords:** hyaluronan, hydrogels, esterification, crosslinking, amphiphiles

## Abstract

In this work, a new amphiphilic derivative made of 10-undecylenic acid grafted to hyaluronan was prepared by mixed anhydrides. The reaction conditions were optimized, and the effect of the molecular weight (Mw), reaction time, and the molar ratio of reagents was explored. Using this methodology, a degree of substitution up to 50% can be obtained. The viscosity of the conjugate can be controlled by varying the substitution degree. The physicochemical characterization of the modified hyaluronan was performed by infrared spectroscopy, Nuclear Magnetic Resonance, Size-Exclusion Chromatography combined with Multiangle Laser Light Scattering (SEC-MALLS), and rheology. The low proton motility and self-aggregation of the amphiphilic conjugate produced overestimation of the degree of substitution. Thus, a novel method using proton NMR was developed. Encapsulation of model hydrophobic guest molecules, coenzyme Q10, curcumin, and α-tocopherol into the micellar core was also investigated by solvent evaporation. HA-UDA amphiphiles were also shown to self-assemble into spherical nanostructures (about 300 nm) in water as established by dynamic light scattering. Furthermore, HA-UDA was crosslinked via radical polymerization mediated by ammonium persulphate (APS/TEMED). The cross-linking was also tested by photo-polymerization catalyzed by Irgacure 2959. The presence of the hydrophobic moiety decreases the swelling degree of the prepared hydrogels compared to methacrylated-HA. Here, we report a novel hybrid hyaluronan (HA) hydrogel system of physically encapsulated active compounds and chemical crosslinking for potential applications in drug delivery.

## 1. Introduction

Hydrogels presents widespread applications in different fields related to human health, including pharmaceutical, bioengineering, food industry, medicine, nutrition, and cosmetics. They serve as supporting material for cells during tissue regeneration [1] and as carriers for drug delivery [2,3]. Hydrogels may contain hydrophobic domains or nanodomains, especially of the micellar type [4]. The behavior of hybrid hydrogels with incorporated hydrophobic (nano)domains resemble the behavior of semi-crystalline polymers, which contain both crystalline and amorphous regions [5]. Besides, the creation of hydrophobic domains into the hydrogel results in a change in the hydration (swelling) as well as in the organization of water molecules within the hydrogel structure.

Hyaluronic acid (hyaluronan, HA), a hydrophilic biopolymer, is an attractive building block for the synthesis of hydrogels due to its ubiquitous presence in the human body. HA is a glycosaminoglycan composed of repeating disaccharide units of *N*-acetyl-d-glucosamine (GlcNAc) and d-glucuronic (GlcA), linked by glycosidic bonds ([→4)-β-d-GlcpA-(1→3)-β-d-GlcpNAc-(1→]).

To decrease the hydrophilicity of HA and increase its resilience time in the body, chemical modification is necessary. Injectable hydrogels based on HA are prepared using various chemical and physical crosslinking methods.

One of most common modifications of HA for chemical crosslinking is (i) methacrylation [6,7], which works in water, but its efficiency is significantly lowered by competitive hydrolysis. Crosslinked HA has a wide range of applications, i.e., for 3D bioprinting [8] or microfabrication [9]. On the other hand, physical crosslinked hydrogels formed by hydrophobic interactions, hydrogen bonding, or charge interaction had demonstrated important applications in cancer therapy [10], tumor targeting [11] and visualization [12], or ophthalmology [13]. However, in all these synthetic methodologies, the conditions involve the use of anhydrous solvents, which makes the process expensive and inconvenient. Considering the broad field of applications of HA and its derivatives, the advantages of the mixed anhydrides methodology used for the covalent attachment of fatty acids and sensitive molecules include: (i) Mild reaction conditions for the activation; (ii) the chemical modification of HA is performed in water, thus HA is not converted to its acidic form with concomitant degradation of the backbone; (iii) high substitution degree (up to 50%); (iv) the esterification reaction is usually performed at room temperature; and (v) high purity of the prepared conjugates.

Our previous work involved the preparation of amphiphilic HA with long-chain fatty acids, such as oleic acid [14], linoleic acid [15], and palmitic acid [16], which showed promise to fabricate self-assembled polymeric micelles for skin delivery. Regarding biological properties, monounsaturated fatty acids promoted apoptosis in various types of cancers [17].

10-undecylenic acid (UDA) is used as antifungal therapy and a nutritional supplement. UDA is an unsaturated medium chain fatty acid derivative, and is a promising neuroprotective substance for an inhibition of µ-calpain [17]. In addition, chitosan has been engineered to possess anti-bacterial/biofilm and anti-cancer potential by incorporating UDA [18]. From a chemical point of view, the incorporation of an unsaturated fatty acid make the conjugate a very attractive synthon for further functionalization by thiol-ene reaction [19,20], olefin cross-metathesis [21], or cross-linking [22]. Hence, our hypothesis was that the incorporation of UDA can be used for the preparation of a hybrid hydrogel, which can be stabilized by physical and chemical crosslinking. 

Polymeric micelles need to be stabilized for efficient drug targeting to tumors and to sites of inflammation. Thus, prolonged circulation times are needed to prevent premature disintegration. Particularly, core-crosslinking is one the most popular methods to improve the in vivo stability [23]. Keeping the above facts in consideration, the present study focused on the synthesis of a novel bioconjugate based on 10-undecylenic acid (UDA) and HA by the mixed anhydrides methodology.

Furthermore, the purpose of the present study was to describe a method to determine the degree of substitution (DS) of hydrophobized hyaluronic acid (HA) by proton nuclear magnetic resonance (^1^H NMR) spectroscopy. NMR spectroscopy can be used to obtain quantitative information about the chemical structure of HA after chemical modification. Low molecular weight HA derivatives can be easily characterized due to the very low viscosity of the solutions. However, the accuracy of DS determination is strongly limited due to the self-aggregation of amphiphilic HA. The characterization was completed by infrared spectroscopy (FT-IR), SEC-MALLS (size exclusion chromatography coupled with a multiangle light scattering system), and gas chromatography (GC). 

Furthermore, the ability of HA-UDA to form dimensionally stable hydrogels was proven by using different initiators.

## 2. Materials and Methods

### 2.1. Materials 

Hyaluronic acid (HA) was obtained from Contipro a.s (Dolni Dobrouc, Czech Republic) Particularly, HA of weight-average molecular weight and polydispersity (in brackets) (Mw = 15,000 [PDI = 1.5], 110,000 g/mol [PDI = 1.7], and 240,000 g/mol, [PDI = 1.8], respectively). Tetrahydrofurane (THF, 99.5%), isopropanol (IPA, 99.7%) were purchased from Lach-Ner (Neratovice, Czech Republic). Benzoyl chloride (BC, 99%), triethylamine (TEA, 98%), 10-undecylenic acid (98%), *N,N*-Diisopropylethylamine (DIPEA), 2-Hydroxy-4′-(2-hydroxyethoxy)-2-methylpropiophenone (Irgacure 2959), ammonium persulphate (APS), *N,N,N′,N′*-Tetramethylethylenediamine (TEMED, ~99%) and 2,2-dimethoxyphenylacetophenone (DMPA, Irgacure 651), riboflavin (98%), undecanoic acid, formic acid, sodium hydroxide, coenzyme Q10, α-Tocopherol, and curcumin were purchased from Sigma-Aldrich (Prague, Czech Republic). Deuterium oxide (D_2_O) was purchased from CortecNet (Voisins le Bretonneux, France). 4-(dimethylamino) pyridine (DMAP, 99.5%) was obtained from Merck (Prague, Czech Republic). 3-(4,5-dimethylthiazol-2-yl)-2,5-diphenyltetrazolium bromide (MTT) and ethidium homodimer were purchased from Life Technologies (Carlsbad, CA, USA).

### 2.2. General Procedure for the Synthesis of Undecenoyl-Hyaluronan 

HA (5.0 g, 12.5 mmol) was dissolved in 100 mL of demineralized water. To that solution, THF (50 mL) was slowly added. After the solution was homogeneous, DIPEA (4.3 mL, 25 mmol, 2 eq. (eq. stands for molar equivalents in ratio to HA dimer) and DMAP (78.6 mg, 0.6 mmol or 0.5 eq. to HA dimer) were consecutively added. The formation of the mixed anhydride was carried out for 60 min at 5 °C. The equivalents of the mixed anhydride used in the reaction feed were varied according to the molar equivalents described in Table 1. Thus, the respective molar eq. of 10-undecenoic acid was dissolved in tetrahydrofurane (50 mL). After that, DIPEA (2 eq. to mixed anhydride) and benzoyl chloride (1 eq. to UDA) were added. Then, the mixed anhydride was added to the solution containing HA. The esterification reaction was carried for 3 h at the same temperature of the activation reaction. The crude product was isolated by precipitation after the addition of 3.65 g of sodium chloride. The product was washed out by adding 1000 mL of IPA. The product was washed with IPA: H_2_O (80% v/v, 4 × 500 mL) and washed three more times with 500 mL of IPA. The white precipitate was decanted and dried in an oven at 40 °C for at least 24 h. The yield (Y) was determined using the modified and non-modified dimers and was calculated using Equation (1), with an average of at least three independent batches for each reaction feed:

(1) $$\mathrm{Yield}\;\left[\%\right]=\;100\times \frac{\frac{m_{\mathrm{product}}\;\left[g\right]}{M_{\mathrm{HANa}}+\left\{M_{\mathrm{Undec}}-M_{H_{2}O}\right\}\;\frac{\mathrm{DS}\;\left[\%\right]}{100}}}{\frac{m_{\mathrm{starting}}\;\left[g\right]}{M_{\mathrm{HANa}}}},$$

where m_starting_ is the amount of HA used in the reaction feed (in grams), m_product_ is the mass of the isolated product, M_Undec_ is the molecular weight of 10-undecenoic acid (184.28 g/mol) and M_HANa_ is 401.3 (g/mol) represents the molecular weight of HA dimer, and DS is the degree of modification obtained by gas chromatography.

The reaction conversion (Equation (2)) was calculated using the value of the degree substitution (DS_UNA_) and the stoichiometric ratio of UDA/HA used in the reaction feed (Table 1):

(2) $$\mathrm{Conversion}=\frac{\mathrm{eq}.\mathrm{UDA}\left(\mathrm{DS}\right)}{\mathrm{eq}.\mathrm{HA}}\times 100,$$

where eq. stands for molar equivalents.

### 2.3. Nuclear Magnetic Resonance Spectroscopy 

NMR (Bruker, Rheinstetten, Germany) was used for the confirmation of the chemical structure of the conjugate as well as for the determination of the degree of substitution. In brief, 8 mg of the derivative was solubilized in 800 µL of D_2_O. ^1^H and ^13^C experiments were carried out on a BRUKER Avance^TM^ III 700 MHz. The ^1^H and ^13^C chemical shifts were referenced to 3-(trimethylsilyl)propionic acid sodium salt (TSPA). ^1^H–^13^C HSQC spectra were acquired using gradient pulse sequences and 2 kHz data points, 80 scans per increment, 256 increments, and heteronuclear scalar coupling C–H set at 145 Hz. DOSY (diffusion ordered spectra) were obtained using a stimulated echo pulse sequence with bipolar gradients (STEBPGP). The degree of substitution (DS) is the (average) number of linked fatty acid moieties (UDA) per 100 disaccharide units on the HA molecule. A sodium hydroxide solution was added to the NMR cuvette for the determination of the degree of substitution. The DS of the derivatives (Figure S3) was determined form the relative integral value of the methylene signal at 2.4 ppm (corresponding to the –CH_2_ in position α to the carbonyl by setting the integral of anomeric CH signals of HA resonating at 4.4 to 4.6 ppm to 100 (in the case of 100% substitution).

### 2.4. Determination of Degree of Substitution of the Degree of Substitution by Gas Chromatography (GC)

To evaluate the free undecylenic acid content, HA-UDA (concentration 1 mg/mL) was dissolved in 50% aqueous solution of 2-propanol/water. Then, the solution was extracted by (9:1) solution of chloroform/hexanes. To determine the amount of total fatty acids, the solution was hydrolyzed by adding a solution of 1% (w/v) of sodium hydroxide at room temperature for 24 h. After the hydrolysis, the solution was neutralized by 2 M formic acid. Finally, the solution was extracted again by (9:1) solution of chloroform/hexanes. The extracts were analyzed using gas chromatography with online derivatization of the fatty acids to the respective trimethylsilyl esters. The analysis was performed on a 7890B gas chromatograph equipped with a 7693 ALS sampler and a flame ionization detector (Agilent Technologies, Waldbronn, Germany). The samples were injected onto a HP-5 ms UI capillary column (30 m × 0.250 mm × 0.25 μm) with He as the carrier gas. The injector temperature was 300 °C, split ratio was 1:10, injected volume was 1 mL of the sample and 0.8 mL of the derivatizing agent, oven temperature was 80 °C (hold 1 min), then 20 °C min^−1^ to 305 °C (hold for 8 min). The detector temperature was 310 °C. Undecanoic acid was used as the internal standard. For calibration, the two series were prepared from a stock solution of the fatty acid in 2-propanol in concentration of 2.5 to 40.0 mg/mL for the determination of the total fatty acid content, and from 0.01 to 0.10 for the free fatty acid content. 

### 2.5. Infrared Spectroscopy

Fourier transform-infrared spectroscopy (FT-IR) spectra were recorded on a FT-IR-8400S spectrometer (Shimadzu, Duisburg, Germany). Samples were studied as KBr pellets using a 64-scan width (between 400 and 4000 cm^−1^), and 2 cm^−1^ resolution.

### 2.6. Determination of Molecular Weight (Mw) by Size-Exclusion Chromatography (SEC)-Multiangle Laser Scattering (MALLS)

Average molecular weight (Mw) and molecular weight distributions were determined by SEC-MALLS using an Agilent degasser Model G 1379A, chromatography system composed of an Agilent HPLC pump Model G 1310A, a Rheodyne manual injector Model 7125i, two 7.8 mm ultrahydrogel Linear columns (Waters), chromatographic detectors included a DAWN EOS MALLS, a ViscoStar differential viscometer, and an Optilab T-rEX differential refractive index in series (Wyatt Technology, Santa Barbara, CA, USA). A refractive index increment (dn/dC) of 0.155 mL·g^−1^ was used for the calculation of the molecular weight and polydispersity (Mw/Mn) of modified HA.

### 2.7. In Vitro Cell Compatibility of the Derivative HA-UDA

Cell viability was investigated for HA-UDA by using NIH-3T3 mouse fibroblast cells. Cell lines were cultured in Dulbecco’s modified eagle’s medium high glucose, 10% heat-inactivated fetal bovine serum (FBS), 100 U·mL^−1^ penicillin, 100 μg·mL^−1^ streptomycin, and 2 mM L-glutamine in a humidified atmosphere of 5% CO_2_ at 37 °C. Untreated cells (without any contact material) were considered as the control. Cell viability was measured 24 to 72 h after treatment using the (3-(4,5-dimethylthiazol-2-yl)-2,5-diphenyltetrazolium bromide) tetrazolium reduction assay (MTT), as described previously [24]. 

### 2.8. Preparation of Polymeric Micelles by Solvent Evaporation

Hydrophobic models were loaded in the formed micelles by thin film-hydration method, following a procedure reported previously by our group [25]. In brief, 6.5 mg of coenzyme Q10, α-Tocopherol, and curcumin were independently dissolved in 1.5 mL of isopropanol and added to 10 mL of a 1%, w/v solution of HA-UDA. The loaded amount was quantified by HPLC. 

The loading capacity and encapsulation efficiency were determined using the following Equations (3) and (4):

(3) $$\mathrm{Loading}\;\mathrm{capacity}\;\left(\mathrm{wt}.\%\right)=\frac{\mathrm{amount}\;\mathrm{of}\;\mathrm{loaded}\;\mathrm{drug}}{\mathrm{amount}\;\mathrm{of}\;\mathrm{loaded}\;\mathrm{polymeric}\;\mathrm{micelles}}\times 100\%,$$

(4) $$\mathrm{Encapsulation}\;\mathrm{efficiency}\;\left(\mathrm{EE}\right)=\frac{\mathrm{amount}\;\mathrm{of}\;\mathrm{loaded}\;\mathrm{drug}}{\mathrm{amount}\;\mathrm{of}\;\mathrm{feeding}\;\mathrm{drug}}\times 100.$$

The loading experiments were repeated at least three independent times.

### 2.9. Dynamic Light Scattering (DLS) Analysis

The micelle size (hydrodynamic diameter) was measured by dynamic light scattering (DLS) using a Zetasizer Nano-ZS Malvern Instruments, Malvern, United Kingdom) at a wavelength of 632.8 nm and a 173° detection angle using back-scattering detection. The micelles were diluted with deionized water or 0.1% (w/v of NaCl) until the concentration provided the light scattering intensity (c = 1 mg·mL^−1^). Three samples from the same formulation were measured once to determine the particle size distribution.

### 2.10. Rheological Characterization

The rheological measurements were performed by a Malvern Kinexus pro+ rheometer (Malvern Instruments, UK) using cone-plate geometry with a 1° angle and 60-mm diameter. The steady state flow experiments were executed in the range of shear rates between 10^−3^ and 104 s^−1^ at 25 °C. Zero shear viscosities, η0, were evaluated from the Newtonian response or using the cross model [26].

### 2.11. Radical Polymerization Mediated by APS/TEMED

A solution of HA-UDA (various DS) was prepared in distilled water using the weight concentration (wt.%) resumed in Table S1. The solution was thoroughly degassed before the reaction. Afterward, the aqueous solution of APS (5.5–66 mmol/L) and TEMED (5.5–75 mmol/L) were added to the HA solution and vortexed to ensure homogenization. The total volume of the solution was kept constant. A new set of reactions was carried by using riboflavin in the concentration of 5.9 to 0.74 mmol/L. The polymerization reaction was monitored by the vial tilting method [27]. 

### 2.12. Photo-Polymerization Mediated by Irgacure

A solution of HA-UDA (or MeHA) was prepared in distilled water using a final concentration of 1.5 to 7.5 wt.%. A solution of isopropanol/water (4:1 v/v) was used for the experiments using DMPA. The photoinitiator Irgacure 2959 or DMPA was used in a concentration of 0.1% (w/v). The cross-linking solution was transferred to Teflon moulds (cylinders, diameter 10 mm, height 5 mm. The reactions were performed in a UV cross-linker CL1000M (Analytik, Jena, Germany), equipped with a chamber (P = 6.75 mW·cm^−2^, E = 12,150 mJ·cm^−2^). The solutions were exposed to 302 nm UV in a range from 1 to 15 min. (Table S2). The hydrogels were released from the mold and washed with distilled water.

### 2.13. Scanning Electron Microscopy (SEM)

The hydrogels were dialyzed and freeze-dried for scanning electronic microscopy analysis (Zeiss Ultra Plus, Oberkochen, Germany). This microscope had a resolution of 0.8 nm and a beam voltage of 0.1 to 30 kV. The samples were analyzed after whipping in a Mini Sputter Coater SC7620 (Quorum Technologies Ltd, Laughton, East Sussex, UK) by gold in approximately a 10 to 20 nm thickness. The samples were measured at 25 °C using the SE2 detector and processed by software SmartSEM V05.06 (1 kV, aperture: 15 μm, magnitude 100–500×).

### 2.14. Determination of Mass Swelling Ratio (Qm)

The mass swelling ratio (Qm) was determined at 25° after 24 h in phosphates buffer (0.1 mol/L, pH 7.4). Qm was calculated according to the following equation:

(5) $$\mathrm{Qm}\;\left(\%\right)=\left(\frac{\mathrm{Ws}-\mathrm{Wd}}{\mathrm{Ws}}\right)\times 100.$$

The result is an average of six determinations (mean ± SD; n = 6); wherein the weight of swollen gel is identified as (Ws) and the weight of the dry gel is identified as (Wd).

## 3. Results and Discussion

### 3.1. Chemical Modification of Hyaluronic Acid Mediated by Mixed Anhydrides

In this work, 10-undecenoic acid (UDA) was activated by benzoyl chloride forming a mixed anhydride (MA) in THF. The activation reaction can be performed in the presence of tertiary amines, i.e., TEA or DIPEA in isopropyl alcohol or THF. After repeated washing of the product, triethylamine was detected in the product as impurity. Thus, the methodology of esterification was studied using DIPEA. To ensure a complete homogenization of the mixed anhydride in the reaction feed, the solvent was mixed with HA solution. The formation of the mixed aliphatic-aromatic anhydride was followed by ^1^H NMR (Figures S1 and S2). The experimental results demonstrated that the intermediate is more stable at 5 °C.

**Figure 1:**
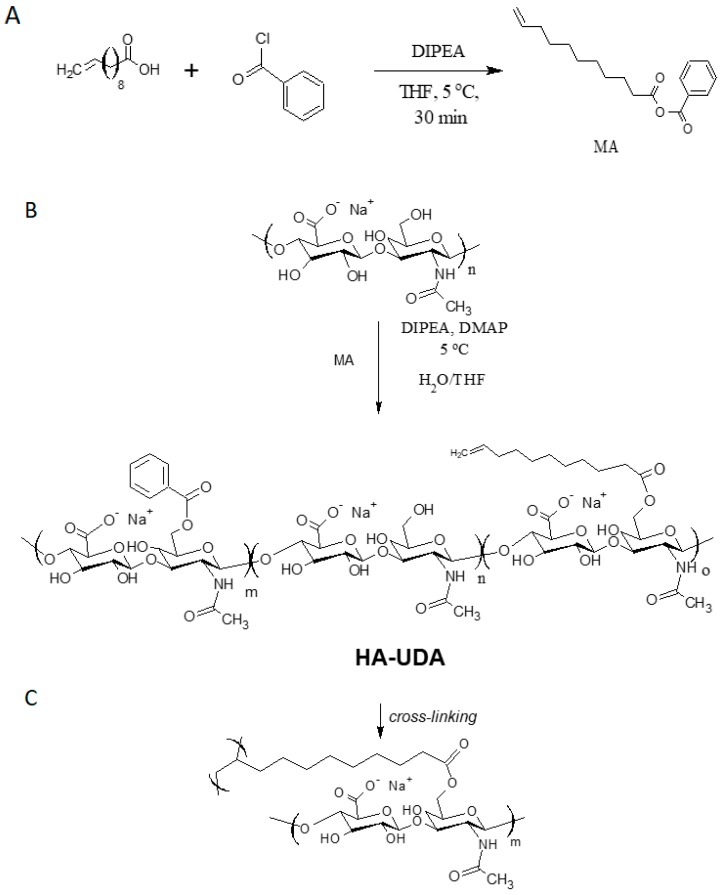
(**A**) The activation of 10-undecenoic acid was mediated by benzoyl chloride and formed the mixed anhydride (MA) (**B**) The acylation of hyaluronic acid was catalyzed by dimethylamine pyridine, and (**C**) denotes the chemical crosslinking of HA-UDA.

In a second step, the mixed anhydride reacts with hydroxyl moieties in HA, yielding esterification (Figure 1). The mixed anhydrides methodology worked for the chemical modification of low and high molecular weight HA. In this work, we focalized in the chemical modification of 15,000 g/mol (LMW-HA) and 240,000 g/mol (HMW-HA) due to the envisioned applications in drug delivery and hydrogels formation, respectively. 

To study the effect of the mixed anhydride (MA) on the degree of substitution, the molar ratio between MA to HA dimer was varied from 0.2:1 (20%) to 1.3:1 (130%). The highest DS was obtained using 130% of the molar ratio of the anhydride (Table 1, entries 4, 9, 16). A higher molar ratio than 130% produced derivatives insoluble in water, which are not useful for the formation of hydrogels (data not shown). The degree of substitution can be controlled by varying the ratio of HA and mixed anhydride. The purity of the conjugates was determined by measurement of ash and dry matter (data not shown). Moreover, the amount of free fatty acid determined by GC was negligible (between 0.03 to 0.5 wt.%). 

### 3.2. Structural Characterization of the Conjugate HA-UDA

**Figure 2:**
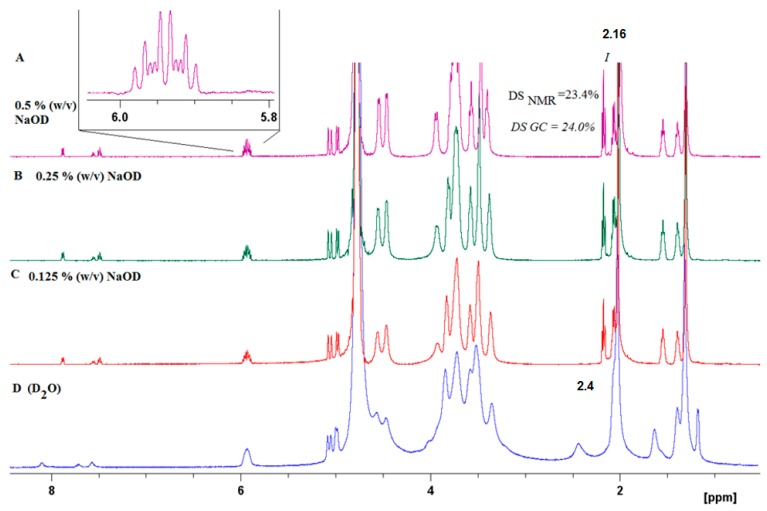
^1^H NMR of HA-UDA (Mw = 261,800 g/mol) acquired in D_2_O without (**D**), and (**A**–**C**) increasing the concentration of NaOD (up to 0.5 % (w/v)).

The success of the covalent grafting was evaluated by combining several analytical techniques, i.e., 1D, 2D NMR, GC, and IR spectroscopy. The physical and chemical properties of HA crosslinked materials depend on the total degree of substitution (DS). Thus, it is necessary to develop an accurate and fast method to determine the DS of modified hyaluronic acid (HA) by proton nuclear magnetic resonance (^1^H NMR) spectroscopy. The influence of the effect of pH on the ^1^H NMR spectra of HA-UDA was studied (Figure S4). Amphiphilic HA usually present an overestimation due to the self-aggregation and restricted mobility of the acyl chains [28]. Particularly, sample concentrations of >10 mg/mL for polyelectrolytes usually produce very viscous solutions or gels, and spectra with broad signals are obtained (see Figure 2D, Figure S5). The best spectra with full proton mobility was obtained when HA-UDA was dissolved in 0.5% (w/v) of NaOD (Figure 2A). After the in-situ hydrolysis of the conjugate by sodium hydroxide, the DS obtained by NMR were in perfect concordance with the values determined by GC (with an average relative deviation of 5%). Recently, Ret et al. demonstrated that the quality of the NMR spectra could be improved by the addition of sodium chloride [29]. In the case of amphiphilic HA, this is not applicable because the presence of salts increases the hydrophobic interactions.

**Figure 3:**
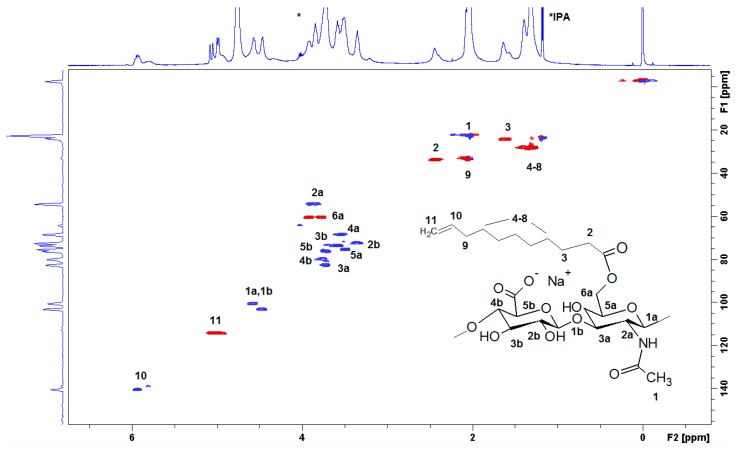
The heteronuclear single quantum correlation (HSQC) spectrum of HA-UDA (15,000 g/mol, DS = 35.4%) recorded in D_2_O. The spectrum was acquired at a concentration of 10 mg·mL^−1^^.^

The full structural assignment of the chemical structure was obtained by HSQC (Figure 3). HA presents anomeric signals at 4.8 to 4.4 ppm (**1a** and **1b**), and skeletal H signals (at 4.2 to 3.2 ppm). Finally, the methyl of N-acetyl group is located at 2.1 ppm (**1**), which is overlapped with the bridge methylene **9** in D_2_O. The DS was determined using the relative integral value of the methylene signal at 2.4 ppm (**2**, –CH_2_ in position α to the carbonyl), which is shifted to 2.2 ppm in NaOD. The β proton was located at 1.6 ppm (**3**) while the additional methylenes appeared at 1.39 (protons **4**–**8**), and the terminal methylidene (**11**) appeared at 4.9 ppm, and the methine (**10**) was located at 5.9 ppm. The benzoylation of HA was also presented (signals at 7,6, 7.7, and 8.0 ppm, respectively). 

**Figure 4:**
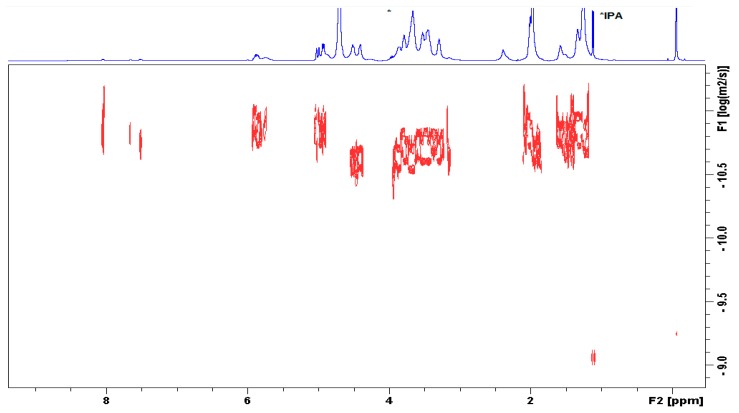
Diffusion-ordered spectroscopy (DOSY) spectrum of HA-UDA determined in D_2_O (HA-UDA, 15 kDa and DS = 35.4%), containing isopropanol as impurity.

The characterization of the conjugate was further established by diffusion ordered NMR spectroscopy (DOSY). Figure 4 shows that the signals of methylenes in UDA, anomeric H-atoms from the HA chain, skeletal H-atoms, and CH_3_ groups from the acetamido substituent lie on one line, i.e., display the same diffusion coefficient. The terminal methylidene, methine, and benzoyl-moieties appeared with slower diffusion due to self-assembly of the derivative. Thus, the aggregated alkyl chains apparently presented a larger molecular size. This observation implies that UDA is covalently linked to the HA polymer.

Finally, the esterification of HA was confirmed by infrared spectroscopy with the band at 1731 cm^−1^ (Figure S6). Additionally, the spectrum shows two sharp bands at approximately 2926 and 2850 cm^−1^, respectively, and assigned to the asymmetrical and symmetrical stretching vibrations of methylene groups, ν*_a_*(CH_2_), and ν*_s_*(CH_2_). Thus, the analysis of FTIR spectra showed that both UDA and HA are present in the final conjugate. However, since the conjugate contains 70% of HA and 40% of UDA in the macromolecule, the most prominent vibration bands on the spectrum are those of HA (Figure S7) [25].

The molecular weight of the modified polysaccharide (Table 1, Mw^2^) is slightly increased due to hydrophobic aggregation. The experimental results demonstrated that the mild conditions used during the esterification reaction have not degraded the HA backbone.

### 3.3. Rheological Characterization

**Figure 5:**
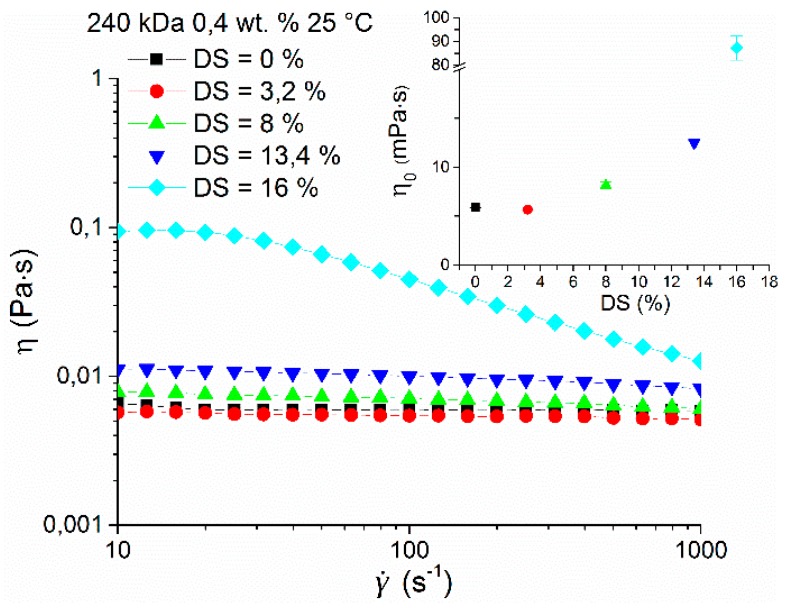
Dependence of viscosity on the shear rate of HA-UDA (240 kDa) with a concentration of 0.4 wt.% and various degree of substitution (DS) in physiological solution (0.9 wt.% of NaCl) at 25 °C. The inset shows the dependence of the determined zero shear viscosities on DS. The graph contains a break between 20 and 80 mPa∙s for better clarity.

The rheological properties of hyaluronan and its esterified derivative HA-UDA were compared in physiological solution (0.9 wt.% of NaCl) at a concentration of 0.4 wt.% and 25 °C (Figure 5). No increase of zero shear viscosity was found at the lowest DS of 3.2% followed by gradual growth up to DS of 16% when a significant increase of one order of magnitude was observed. At low DS, the results suggested that the derivatives form small and weak aggregates that are accessible to hydrophilic solvent. At low concentration and low substitution degrees, intramolecular interactions prevail over aggregation. The last fact would explain a very slight decrease of the viscosity of the sample with DS equal to 3.2. However, the difference between the native HA and 3.2% substituted HA-UDA is very close to the experimental uncertainty to make any further interpretation.

At DS of 16% and higher, the behavior can be attributed to the gradual growth of nanostructures via intermolecular hydrophobic interactions, resulting in well-developed aggregates, with the hydrophobic core inaccessible to the hydrophilic solvent. The compactness of nanostructures is further supported by significant shear-thinning behavior of this sample caused by structure breakdown with increasing shear rate (above 30 s^−1^), similar to that reported before for amphiphilic polysaccharides [30]. Maximum aggregation of the amphiphilic HA was determined for concentrations close to the critical concentration between the dilute and semi-dilute regime (C_cr_), for DS between 12% to 35%, which corresponds to the range where nanostructured aggregate formation was found.

### 3.4. Drug Loading in HA-UDA

Next, the self-assembly capabilities of HA-UDA nanogels were tested. To meet these requirements, low molecular weight HA (15 kDa) was modified. The degree of substitution of HA-UDA was calculated to be 9.4% and 32.6%. Three hydrophobic models, coenzyme Q_10_, α-tocopherol, and curcumin, were independently loaded in the derivative. Coenzyme Q_10_ (2,3-dimethoxy-5-methyl-6-decaprenyl-1,4-benzoquinone, Q_10_) is a fat-soluble hydrophobic compound used broadly in skincare and cosmetics [31] and age-related chronic conditions [32]. The second model, α-tocopherol, degrades rapidly by exposition to light, heat, or oxygen [33]. Curcumin (CUR), the third model, is an anti-psoriatic drug that is insoluble in water [34]. Table 2 summarizes the loading capacity (LC) and encapsulation efficiency (EE). LC and EE were calculated according to Equations (3) and (4). HA-UDA was able to encapsulate a high amount of α-tocopherol, and a lower amount of the aromatic hydrophobic models. The data indicated that of α-tocopherol was structurally more compatible with HA-UDA due to hydrophobic interactions between the alkyl chains of the tocopheryl/undecanoyl substituents, similar to previously reported results [35]. 

The hydrodynamic diameters for HA-UDA-loaded micelles with CoQ10, CUR, and α-tocopherol were around 300 ± 100 nm when dispersed in water. Additionally, the micelles presented a binomial distribution with a size of 270 ± 97 nm (82%) in PBS and 44.6 ± 10 nm (18.1%). The average hydrodynamic size of the micelles loaded with α-tocopherol was included in Figures S9 and S10.

### 3.5. Determination of Cell Viability after Chemical Modification

**Figure 6:**
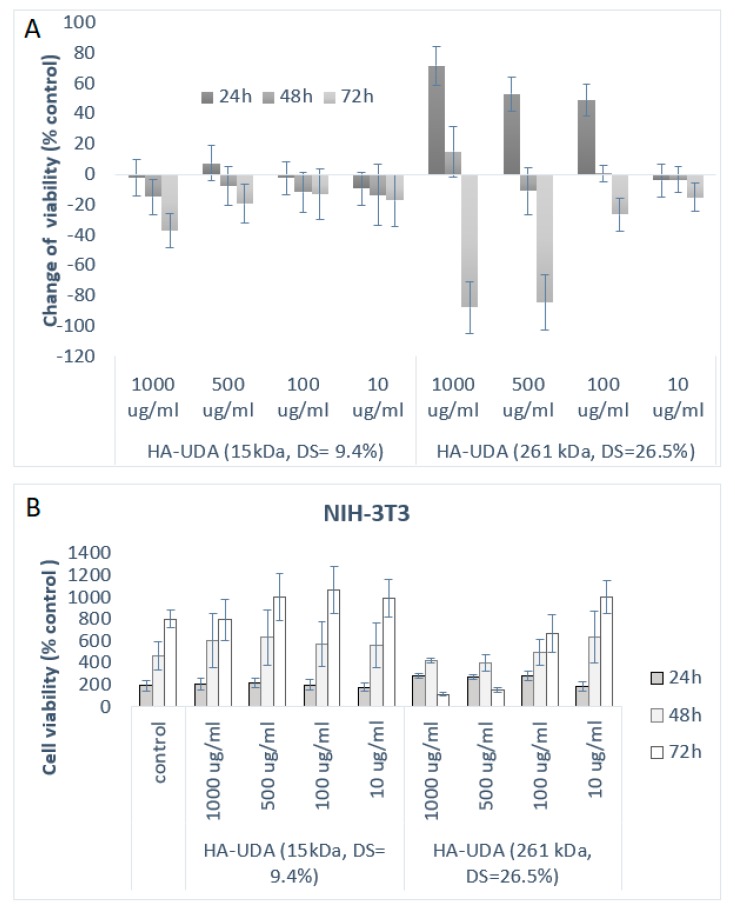
(**A**) Inhibitory effects of HA-UDA are expressed as the change of viability (%) and represent the mean ± SEM of four individual experiments as a function of time (**B**) and cell viability in respect to control for HA-UDA.

The cytotoxicity of HA-UDA materials was evaluated by direct contact using (NIH-3T3) cell fibroblasts. The standard cytotoxicity model 3T3 Swiss cell line was chosen as the control cell line because it is nontumorigenic and at the same time its cell cycle is fast [36,37]. As it is shown in Figure 6, HA-UDA positively affected the growth of NIH-3T3 cells after 24 h of incubation. The derivatives have not shown any acute cytotoxicity in the studied concentrations (10–1000 μg/mL). Furthermore, the derivatives slowed down the cell growth after 48 h of incubation time. After 72 h, the derivatives started to be metabolized by the cells and showed a slightly cytotoxic effect. 

### 3.6. Crosslinking of HA-UDA

The focus of this study was to prepare a hybrid hydrogel. Thus, an evaluation of the effects of the initiator towards the chemical crosslinking of the material could be used for the stabilization of polymeric micelles, as well as the stabilization of these assembles enbibed in a hydrogel. 

The viscosity of HA solutions depends clearly on the molecular weight of HA, and concentration of the polymer. In the first screening, the effect of Mw as evaluated (110, 240, and 480 kDa) in the hydrogel’s formation (Table S2). The medium size Mw were found to be the most adequate. To obtain homogeneous hydrogels, the effect of the degree of substitution was also studied (DS 3.2% to 42%). In a different set of reactions, the effect of the concentration was evaluated in the range of 1.5% to 7.5% (w/v).

**Figure 7:**
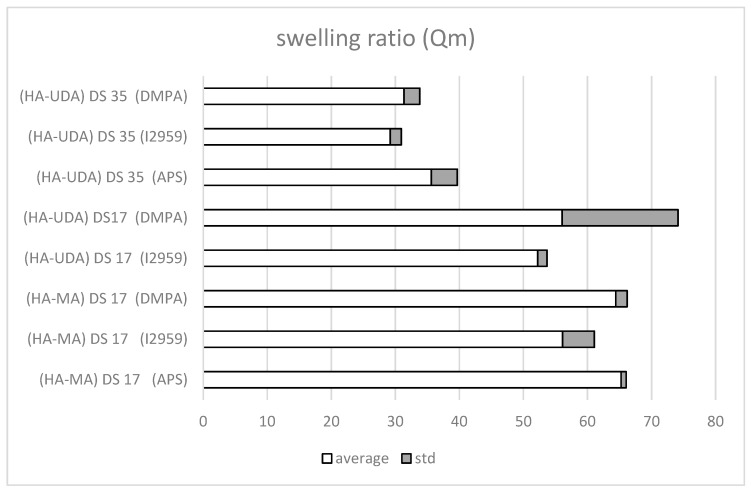
Mass swelling ratio, Qm, of methacrylated-HA (HA-MA) and HA-UDA (Mw = 240,000 g/mol, DS = 17% or 35%, at 1.5 (wt.%). The cross-linking was mediated by APS/TEMED, Irgacure 2959, or DMPA, respectively.

The photoinitiators, 2-hydroxy-1-[4-(2-hydroxyethoxy)phenyl]-2-methyl-1-propanone-1-one (Irgacure 2959) and the more hydrophobic photoinitiator DMPA were tested for UV curing. The equilibrium mass swelling ratio (Qm) indexes the efficiency of the cross-linking (Figure 7). Qm was determined by the ratio of the weight of swollen (Ws) and dry (Wd) gel (Equation (5)).

The photo cross-linking mediated by Irgacure 2959 have occurred after 5 min of irradiation (Table S3), while DMPA requires a longer time (~10 min). Derivatives of high molecular weight HA were used (HA-UDA, Mw = 240,000 g/mol, DS of 17% and 35%). Negligible differences were observed for the swelling ratio for Irgacure 2959 (Qm = 31.4 ± 2.5) and for DMPA (Qm = 29.2 ± 1.8). Moreover, the presence of the hydrophobic moiety reduced the swelling degree of the hydrogel compared to the methacrylated ones.

Furthermore, the free radical initiator system (APS/TEMED reduction–oxidation reaction) was also evaluated due to the observed degradation of active compounds under UV curing (Figure S8). The swelling ratio of HA-UDA showed that DS of 35% was the most efficient. In this case, a low concentration of derivative (1.5 wt.%) was used and a minimum swelling ratio was found (Qm = 35.6 ± 4.07). When the DS was lower (between 3.2% to 26.5%), chemical crosslinking was not observed (gels break in pieces). Physical hydrogels mediated by hydrophobic associations were formed at DS higher than 35%. Then, chemical crosslinking was not necessary. However, this fact has opened the possibility for new applications of the derivative.

**Figure 8:**
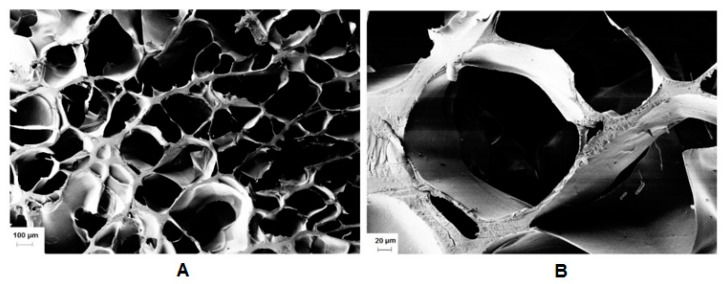
(**A**) SEM cross-sectional view of lyophilized gel obtained by photo crosslinking of HA-UDA (Mw = 240,000 g/mol; DS = 35%, 3.0 wt.%), and (**B**) detailed view of the pores. The cross-linking was mediated by Irgacure 2959 (scale bar: 100 and 20 µm, respectively).

Subsequently, we examined the effect of the molar ratio of the initiator/activator. APS was used in a concentration of 66 to 5.5 mmol/L. The effect of TEMED was evaluated in a concentration from 0 to 75 mmol/L. The use of TEMED led to a significant decrease of gelation time from hours to seconds. However, the experimental results demonstrated that the hydrogel lacked a homogenous structure due to fast gelation. Gelation occurred very fast (in seconds) except in the case of an equimolar ratio of APS/TEMED (11 mmol/L), in which a transparent hydrogel was obtained and the minimum mass swelling ratio. Riboflavin was used to eliminate TEMED, as the oxidative system may exhibit toxicity [38]. The addition of riboflavin also formed a network, which could be a sign that crosslinking could be mediated by visible light [39]. Even though, the most homogeneous materials were produced by Irgacure 2959 (Figure 8). It is worth studying the radical cross-linking for light-sensitive payloads (Figure S9).

## 4. Conclusions

In this work, benzoyl chloride mediated the activation of 10-undecenoic acid that contains electron-rich olefins suitable for further cross-linking or functionalization. The esterification of HA proceeded in very good yields, with a perfect control of the degree of substitution (up to 50%). The conjugate retains the biocompatibility of HA. The presence of undecylenic-long chains caused spontaneous self-aggregation. 

The NMR results clearly showed that low proton mobility significantly overestimated the degree of substitution due to self-aggregation. Variation of pH was a powerful tool to enhance the quality of NMR spectra. Thus, an easy and fast method to estimate the degree of substitution was developed. 

Encapsulation of hydrophobic drugs was also possible due to the amphiphilicity of the derivative. Furthermore, the derivative can be used for the preparation of 3D crosslinked networks. Notably, Irgacure 2959-mediated photopolymerization produced structurally homogenous hydrogels characterized by a honeycomb structure, high porosity, and large pores. The swelling degree of the HA-UDA hydrogel was lower than the hydrogels made of methacrylic HA due to the presence of the hydrophobic alkyl chains. Thus, the resulting materials contained hydrophobic blocks, which create strong hydrophobic associations in water. This derivative represents a highly potential for the preparation of hybrid hydrogels stabilized by physical and chemical crosslinking. 

## 5. Patents

C6-C18-Acylated derivative of hyaluronic acid, method of preparation thereof, nanomicellar composition on its based, method of preparation thereof, Šmejkalová, Huerta et al. 2014 (EP 2 934 592 B1).

## Figures and Tables

**Table 1:** Optimization of the esterification reaction for the preparation of Undecenoyl-Hyaluronan (HA-UDA) by varying the molar ratio of mixed anhydride.

Entry	Mw^1^	Mw^2^ (PDI) ^a,b^	MA/HA ^c^ (%)	DS_UDA_^d^ (%)	DS_(Bz)_^e^	Yield ^f^ (%)
1	15	16.4 (1.3)	30	9.4 ± 0.18	0.5 ± 0.1	94.5
2		15.8 (1.3)	50	16.0 ± 2.1	1.6 ± 0.5	96.7
3		13.6 (1.1)	100	32.3 ± 2.1	2.5 ± 0.05	99.5
4		16.5 (1.2)	130	35.9 ± 5.0	3.6 ± 0.05	98.9
5	110	119.8 (1.4)	30	8.1 ± 0.2	0.5 ± 0.05	95.7
6		117.8 (1.5)	50	16.1 ± 2.0	1.7 ± 0.1	98.3
7		125.1 (1.6)	70	20.3 ± 2.5	2.2 ± 0.1	98.8
8		125.4 (1.3)	100	32.4 ± 4.8	3.0 ± 0.2	98.0
9		nd	130	51.8 ± 5.0	5.0 ± 0.5	95.0
10	240	232.9 (1.6)	15	3.2 ± 0.5	0.2 ± 0.1	89.6
11		225.4 (1.7)	20	4.7 ± 0.5	0.3 ± 0.1	96.5
12		224.2 (1.6)	30	8.2 ± 0.3	0.5 ± 0.1	94.8
13		195.5 (1.7)	50	17.2 ± 1.0	1.7 ± 0.5	91.4
14		261.8 (1.5)	90	26.5 ± 2.3	2.0 ± 0.3	92.4
15		268.4 (1.5)	100	32.8 ± 3.0	3.5 ± 0.2	92.4
16		nd	130	42.0 ± 2.5	3.4 ± 0.2	99.6

^a^ The average Molecular weight of HA before the chemical modification (Mw^1^) and the conjugate (Mw^2^) were determined by SEC-MALLS (see Table S1). The polydispersity is included in brackets; ^b^ nd = non-determined, which means that the high degree of substitution produces physical crosslinking and the conjugate interacts with the chromatographic column impairing the determination. ^c^ The mixed anhydride (MA) is generated via the activation of UDA by benzoyl chloride (Figure 1). ^d^ The degree of esterification of undecenoyl-moieties was determined by gas chromatography. ^e^ The degree of substitution of the benzoylated (Bz) ester of HA was determined by gas chromatography. ^f^ The mixed anhydride reacts with HA for 3 h at 5 °C. All the reactions were performed using 2.5% (w/v).

**Table 2:** Encapsulation of hydrophobic models in amphiphilic hyaluronan using curcumin, coenzyme Q10 (coQ10), and α-tocopherol models. Values are presented as mean ± SD; *n* = 3.

DS_GC_ (%)	α-Tocopherol		Co(Q10)		Curcumin	
	Drug Loading % (wt./wt.)	EE (%)	Drug Loading % (wt./wt.)	EE (%)	Drug Loading %	EE (%)
8.2	15.12 ± 0.22	75.2 ± 0.4	6.73 ± 0.25	40.8 ± 0.8	0.09 ± 0.01	25.5
27.8	14.96 ± 0.05	74.9 ± 0.6	7.46 ± 0.42	57.7 ± 0.3	0.14 ± 0.01	35.5

## Supplementary Materials

The following are available online at https://www.mdpi.com/2073-4360/12/1/35/s1, Figure S1: The formation of the mixed anhydride in isopropanol-d8 occurs at 5 °C; Figure S2: reaction of undecylenic acid at (A) room temperature and (B) 5 °C performed in THF d8; Figure S3: Example of integral of HA-UDA; Figure S4: Determination of degree of substitution by hydrolysis of HA-UDA (DS_GC_ = 35%, 240,000 g/mol) using NaOD; Figure S5: Estimation of degree of substitution determined by NMR in D_2_O and sodium hydroxide compared with GC; Figure S6: Infrared spectra of HA-UDA (6,000 g/mol, DS = 46%); Figure S7: Infrared spectra of native HA used for the chemical modification; Figure S8: Encapsulation of Dio/DiI and Nile red in HA-UDA (110,000 g/mol DS = 20%) and cross-linking mediated by Irgacure 2959; Figure S9: Size distribution by intensity for HA-UDA loaded by α-Tocopherol; Figure S10: Size (Z-average and standard deviation) for HA-UDA; Table S1: SEC-MALLS analyses of the HA-samples used for the optimization study; values of weight average molar mass (Mw) and polydispersity index (Mw/Mn) and intrinsic viscosity are reported; Table S2: Gelation time as a function of APS/TEMED or APS/Riboflavin, degree of substitution and concentration of derivative; Table S3: Gelation time as a function of initiator, degree of substitution and concentration of derivative for photo-polymerisation.
