# Supplementary material for: Synthesis and Physicochemical Characterization of Undecylenic Acid Grafted to Hyaluronan for Encapsulation of Antioxidants and Chemical Crosslinking
Source: Polymers (Basel). 2019 Dec 24;12(1):35. doi: 10.3390/polym12010035 (PMC7023664; doi:10.3390/polym12010035)
Supplement: Supplementary file 1 [file polymers-12-00035-s001.pdf]

polymers

MDPI

Article

Synthesis and physicochemical characterization of undecylenic acid grafted to hyaluronan for encapsulation of antioxidants and chemical crosslinking.

Gloria Huerta-Angeles[1*], Martina Brandejsová[1], Kateřina Kopecká[1], František Ondreáš, Tomáš Medek[1], Ondrej Židek, [1] Jaromír Kulhánek[1], Hana Vagnerová[1] and Vladimir Velebný[1]

[1]Contipro a.s., Dolni Dobrouč 401, 561 02 Dolni Dobrouč, Czech Republic
*Correspondence: huerta-angeles@contipro.com; Tel.: +420-465-519-573

S1. Synthesis of methacrylic hyaluronan (MA-HA)

Hyaluronic acid (5.0 g, 12.5mmol of HA dimer) with a mean molecular weight of 280,000 g/mol was dissolved in 100 ml of distilled water. To that solution, THF (100 mL) was slowly added. After the solution was homogeneous, TEA (3.5 mL, 25 mmol) and DMAP (76 mg, 0.63 mmol) and methacrylic anhydride (1.86 mL,12.5 mmol) were consecutively added. The reaction was carried out for 2 hours at 25°C. The product was isolated by precipitation with the addition of 3.65 g of sodium chloride dissolved in 12.5 ml of distilled water. The product was precipitated by addition of 1000 ml of isopropanol (IPA). The product was washed with IPA: H_2O (80% v/v, 4 x 500 ml) and washed three more times with 500 ml of IPA. The white precipitate was decanted and dried in an oven at 40°C for at least 24 hrs. Yield of the reaction: 95% degree of substitution 17%.

S2. Encapsulation of dyes and cross-linking

NR or DiO/Dil loaded micelles were prepared by solvent evaporation method. 1–5 mg of NR dissolved were dissolved in chloroform or DiO/Dil dissolved in isopropanol were added to 10 mL of 1% HA-UDA aqueous solution and intensively stirred for 4 h. The organic solvent was removed by rotary evaporation till a transparent film was achieved. The dried film was further hydrated with 10 mL of water. Non-incorporated (i.e. not dissolved) NR or DiO/Dil was removed by filtration (1mm glass fiber syringe filter) and the resulting filtrate containing polymeric micelles was freeze-dried. After that, 1 % (w/v) solution of the prepared polymeric micelles was crosslinked by Irgacure 2959 mediated by UV.

Table S1. SEC-MALLS analyses of the HA-samples used for the optimization study; values of weight average molar mass (Mw) and polydispersity index (Mw/Mn) and intrinsic viscosity are reported.

Sample	DS_{GC}(%)	attached UDA (%)	Free UDA (% w/w)	DS_{GC2} (%)	attached UDA_2 (%)	Mw (kDa) (PDI)	Dry mass (%)	ash (%)
Batch 1	13.4	12.1	< 0,1	13.4	10.7	223.3 (1.89)	87.29	5.84
Batch 2	16.5	12.8	< 0,1	14.2	10.7	232.7 (1.79)	87.35	6.43
Batch 3	13.8	12.9	< 0,1	13.8	10.9	224.8 (1.85)	86.84	7.13

DS_{GC2}The degree of modification was determined after 6M (accelerated stability at 40°C).

Table S2. Gelation time as a function of APS/TEMED or APS/Riboflavin, degree of substitution and concentration of derivative.

Entry	Mw (kDa)	DS (%)	[C] % (w/v)	APS[a] [mmol/l]	Rib [mmol/l]	TEMED[a] [mmol/l]	Gel. time (min)	swelling
1	240	8.2	5	66.0	0	11	--	No gel
2		17.0					--	No gel
3		24.0					--	No gel
4		26.5	7.5	66.0		11	90	Break in pieces
5			4	16.2	0	11	60	Break in pieces
6			4	16.2	13.9	0	55	Break in pieces
7			3	66.0	0	11	90	Soft gel
8			3	66.0	13.9	0	120	Soft gel
9		*35.8*	3.0	11	0	11	After mixing	inhomogeneous
10			*1.5*	11	0	11	20	Gel

Table S3. Gelation time as a function of initiator, degree of substitution and concentration of derivative for photo-polymerisation.

Entry	M_w	DS	[C]	Gelation time (min)
				**Irgacure I2959 (mmol ml^{-1})**
11	240	35.8	5.5	10
12		17	11	5
				**DMPA (mmol ml^{-1})**
13	240	35.8	5.5	10
14		17	11	12

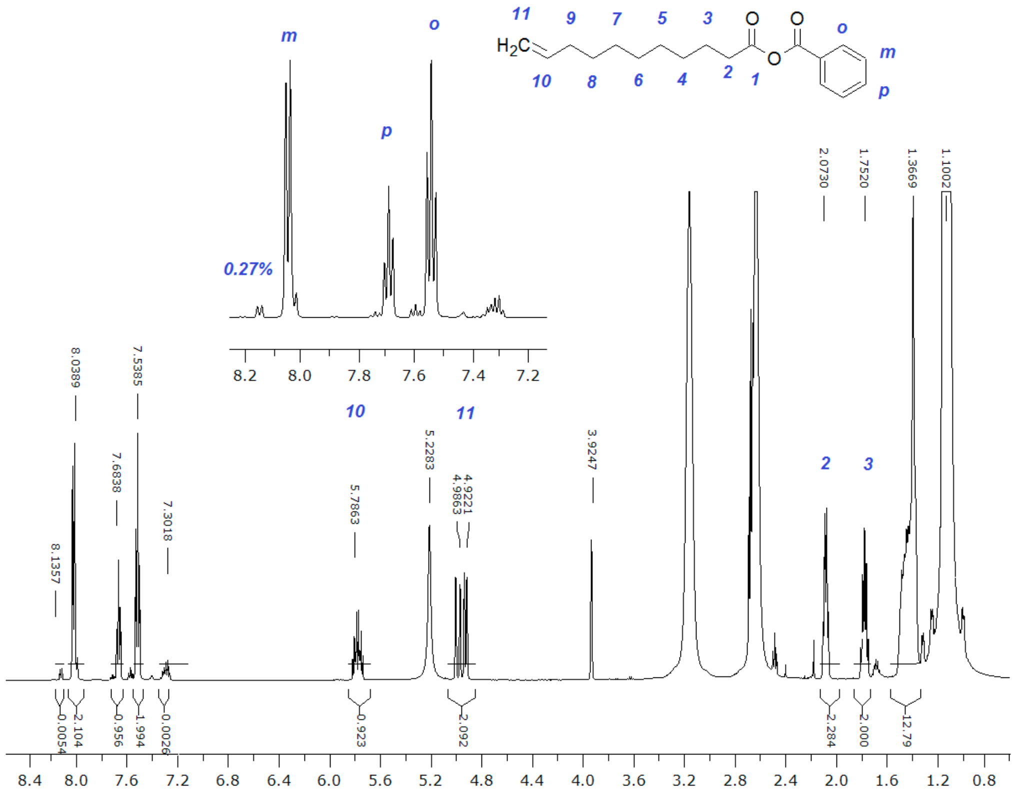

Figure S1. The formation of the mixed anhydride in isopropanol-d8 occurs at 5 °C.

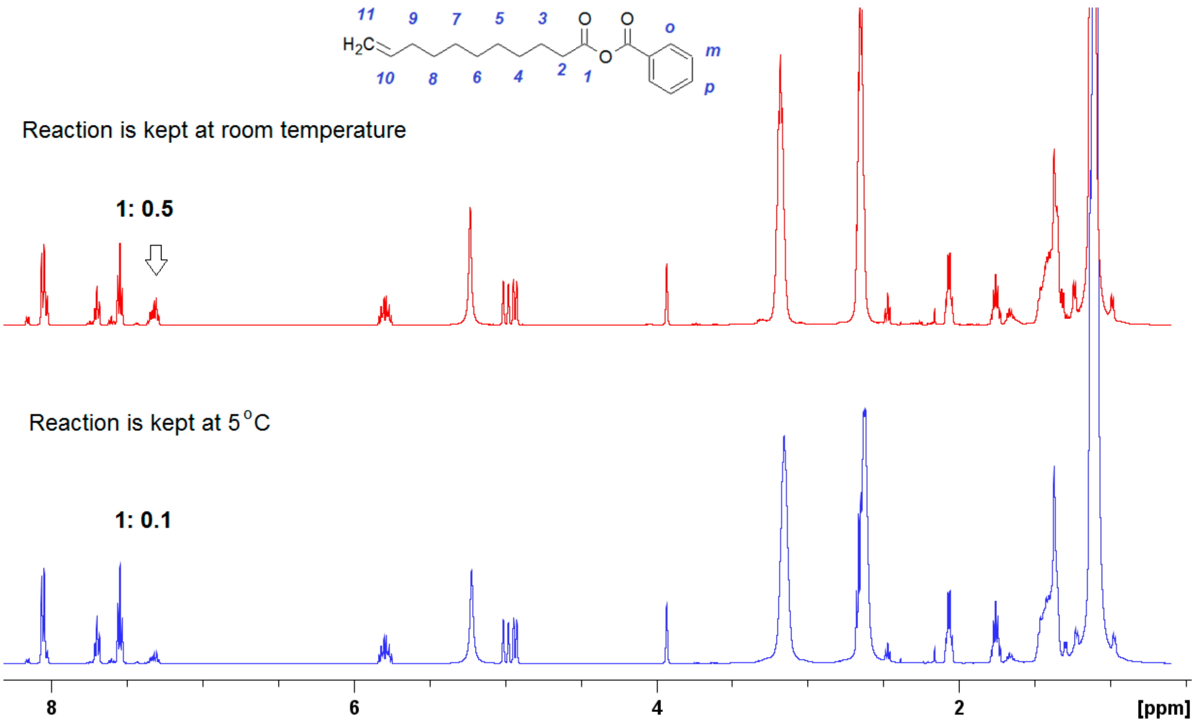

Figure S2. reaction of undecylenic acid at (**A**) room temperature and (**B**) 5 °C performed in THF d8.

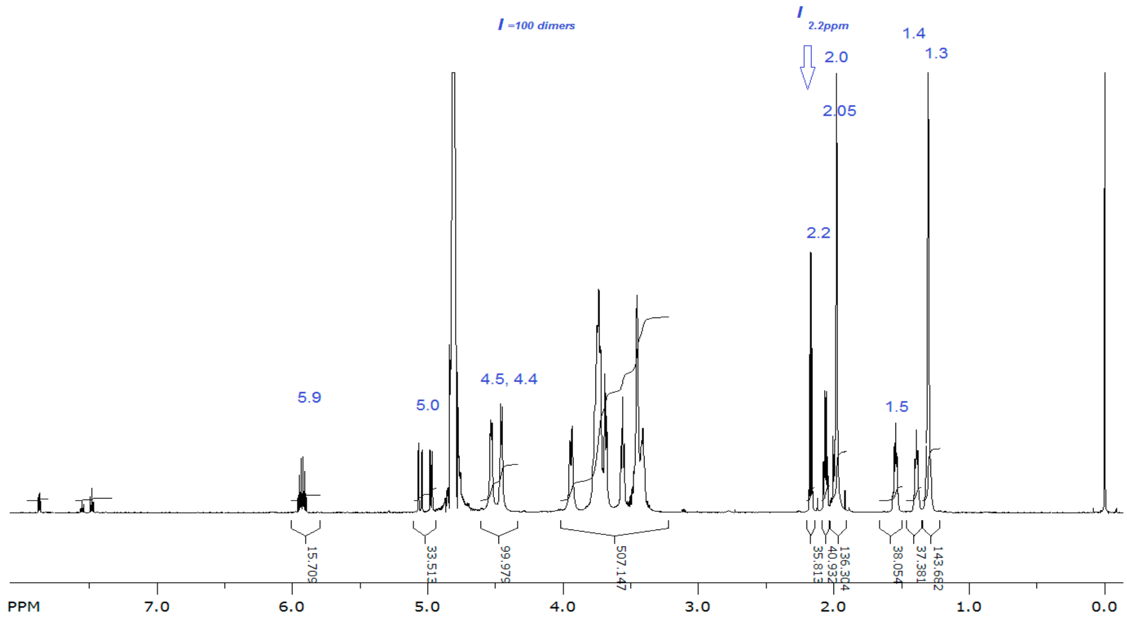

Figure S3. Example of integral of HA-UDA.

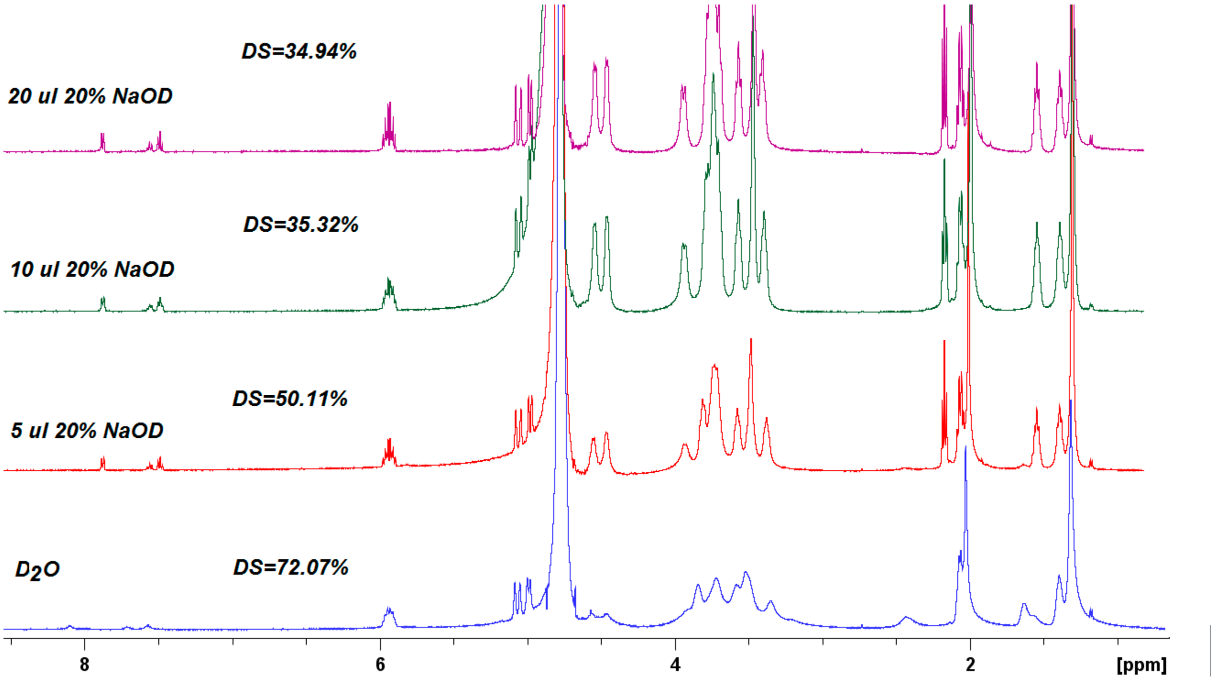

Figure S4. Determination of degree of substitution by hydrolysis **of HA-UDA** (DS_{GC} = 35%, 240,000 g/mol) using NaOD.

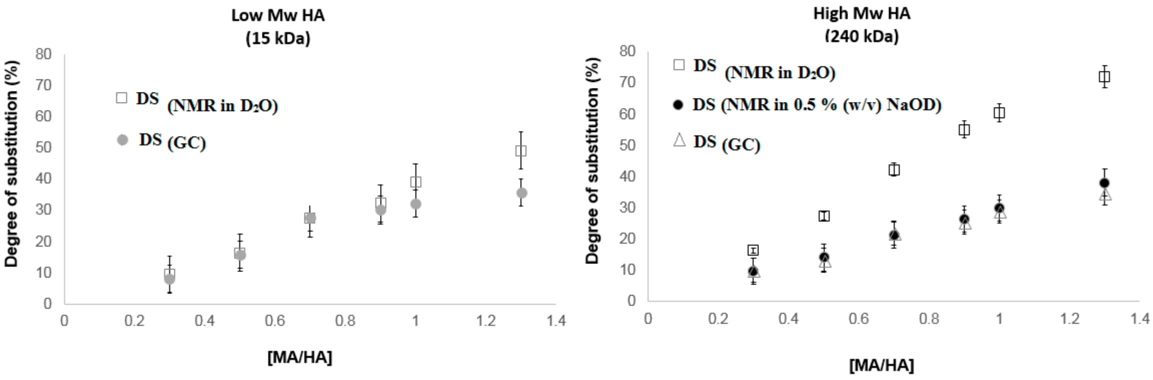

Figure S5. Estimation of degree of substitution determined by NMR in D_2O and sodium hydroxide compared with GC.

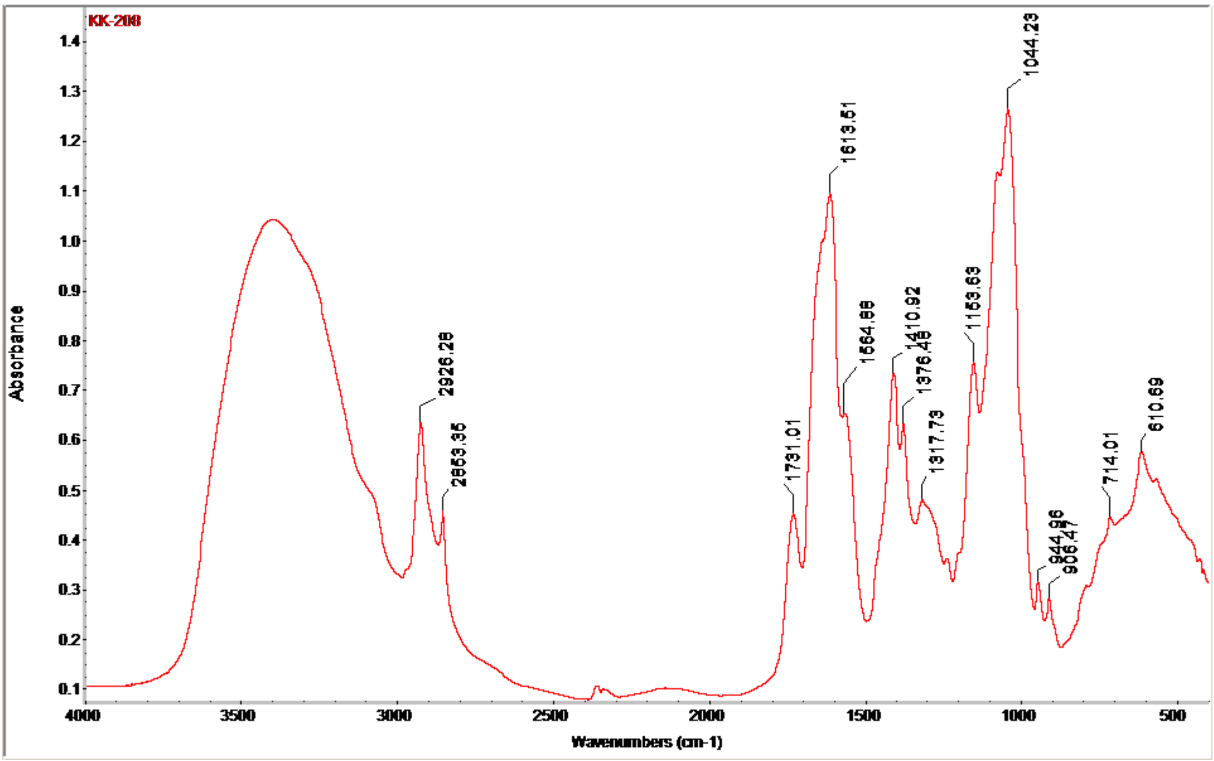

Figure S6. Infrared spectra of HA-UDA (6,000 g/mol, DS = 46%).

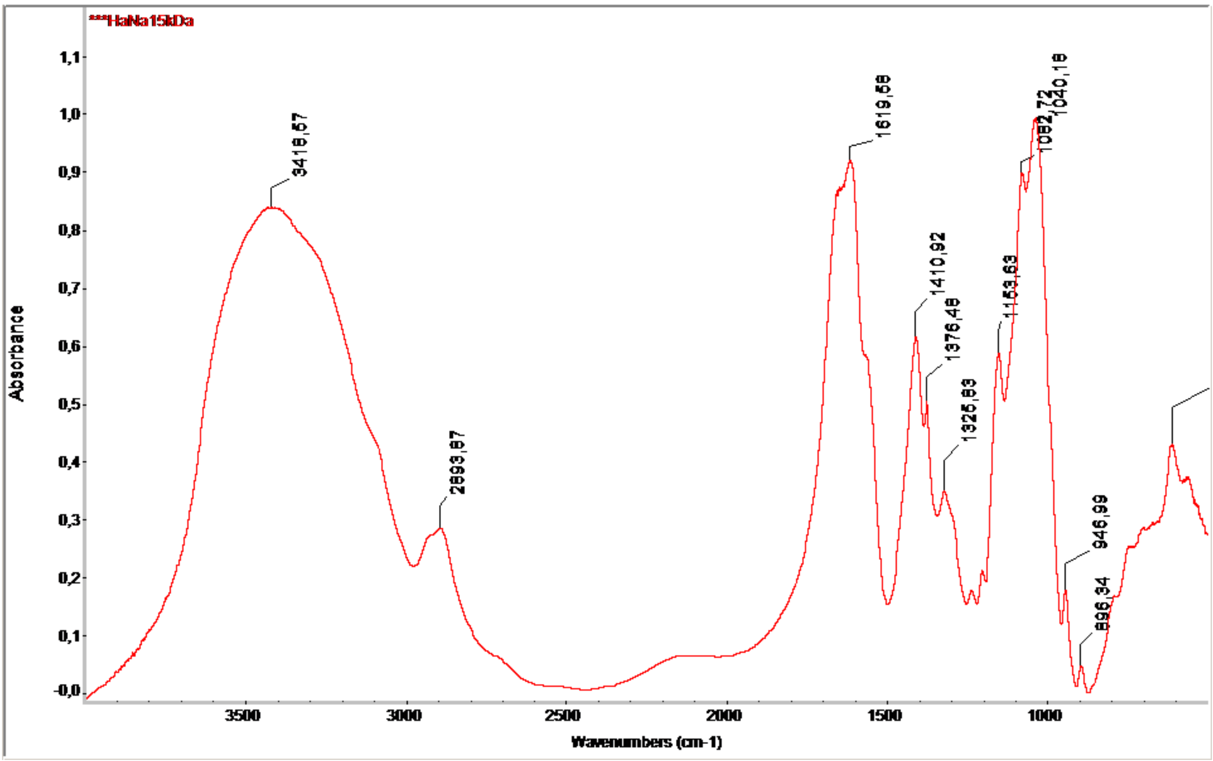

Figure S7. Infrared spectra of native HA used for the chemical modification.

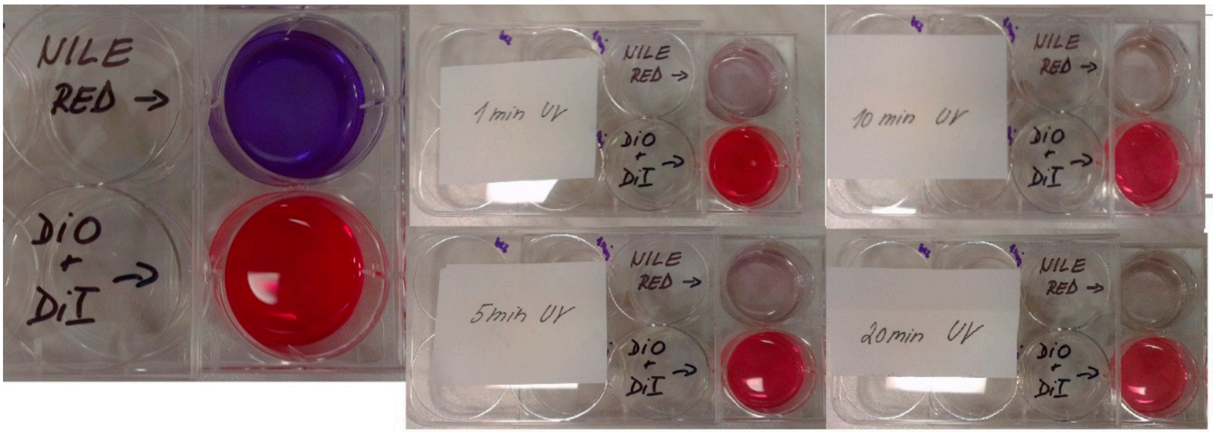

Figure S8. Encapsulation of Dio/DiI and Nile red in HA-UDA (110,000 g/mol DS = 20%) and cross-linking mediated by Irgacure 2959.

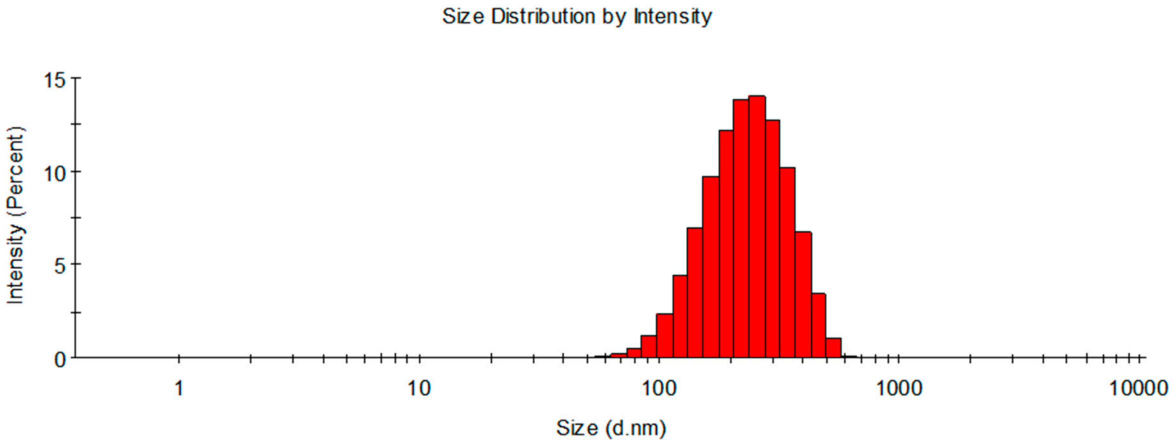

Figure S9. Size distribution by intensity for HA-UDA loaded by α-Tocopherol.

			Size (d.nm):	% Intensity:	St Dev (d.nm):
Z-Average (d.nm):	276,8	Peak 1:	246,8	100,0	94,75
PdI:	0,431	Peak 2:	0,000	0,0	0,000
Intercept:	0,852	Peak 3:	0,000	0,0	0,000
Result quality :	Good				

Figure s10. Size (Z-average and standard deviation) for HA-UDA loaded by α-Tocopherol.

© 2019 by the authors. Submitted for possible open access publication under the terms and conditions of the Creative Commons Attribution (CC BY) license (http://creativecommons.org/licenses/by/4.0/).
